# The predictive accuracy of machine learning for the risk of death in HIV patients: a systematic review and meta-analysis

**DOI:** 10.1186/s12879-024-09368-z

**Published:** 2024-05-06

**Authors:** Yuefei Li, Ying Feng, Qian He, Zhen Ni, Xiaoyuan Hu, Xinhuan Feng, Mingjian Ni

**Affiliations:** 1 Public Health, Xinjiang Medical University, Urumqi, Xinjiang 830011 China; 2 Urumqi Maternal and Child Health Hospital, Urumqi, Xinjiang 830000 China; 3 STD/HIV Prevention and Control Center, Xinjiang Uighur Autonomous Region Center for Disease Control and Prevention, No. 138 Jianquan 1st Street, Tianshan District, Urumqi, Xinjiang 830002 China; 4 Clinical Laboratory, Second People’s Hospital of Yining, Yining, Xinjiang 835000 China

**Keywords:** HIV, Predictive model, Risk of death, Systematic review, Meta-analysis

## Abstract

**Background:**

Early prediction of mortality in individuals with HIV (PWH) has perpetually posed a formidable challenge. With the widespread integration of machine learning into clinical practice, some researchers endeavor to formulate models predicting the mortality risk for PWH. Nevertheless, the diverse timeframes of mortality among PWH and the potential multitude of modeling variables have cast doubt on the efficacy of the current predictive model for HIV-related deaths. To address this, we undertook a systematic review and meta-analysis, aiming to comprehensively assess the utilization of machine learning in the early prediction of HIV-related deaths and furnish evidence-based support for the advancement of artificial intelligence in this domain.

**Methods:**

We systematically combed through the PubMed, Cochrane, Embase, and Web of Science databases on November 25, 2023. To evaluate the bias risk in the original studies included, we employed the Predictive Model Bias Risk Assessment Tool (PROBAST). During the meta-analysis, we conducted subgroup analysis based on survival and non-survival models. Additionally, we utilized meta-regression to explore the influence of death time on the predictive value of the model for HIV-related deaths.

**Results:**

After our comprehensive review, we analyzed a total of 24 pieces of literature, encompassing data from 401,389 individuals diagnosed with HIV. Within this dataset, 23 articles specifically delved into deaths during long-term follow-ups outside hospital settings. The machine learning models applied for predicting these deaths comprised survival models (COX regression) and other non-survival models. The outcomes of the meta-analysis unveiled that within the training set, the c-index for predicting deaths among people with HIV (PWH) using predictive models stands at 0.83 (95% CI: 0.75–0.91). In the validation set, the c-index is slightly lower at 0.81 (95% CI: 0.78–0.85). Notably, the meta-regression analysis demonstrated that neither follow-up time nor the occurrence of death events significantly impacted the performance of the machine learning models.

**Conclusions:**

The study suggests that machine learning is a viable approach for developing non-time-based predictions regarding HIV deaths. Nevertheless, the limited inclusion of original studies necessitates additional multicenter studies for thorough validation.

## Background

AIDS (Acquired Immune Deficiency Syndrome) is a severe infectious disease caused by the human immunodeficiency virus (HIV), leading to a substantial number of global fatalities each year. According to the United Nations Programme on HIV/AIDS (UNAIDS) report as of December 2022, a total of 85.6 million individuals worldwide had contracted HIV, and 40.4 million had succumbed to AIDS-related illnesses since the onset of the epidemic [1]. In the previous year, despite some countries achieving the 95-95-95 target ahead of schedule, there is a worrisome surge in new HIV infection cases in certain countries in Asia and the Pacific region [2]. Particularly in specific resource restrained countries and regions, the persistent prevalence of HIV infection remains a substantial public health concern.

Although the development of antiretroviral treatment (ART) has significantly extended the life expectancy of people with HIV, prior studies indicate that the majority of individuals living with HIV (PWH) experience a shorter survival period compared to their healthy counterparts, and face a heightened risk of death during the infection period. This poses numerous challenges to clinical practice [3, 4]. For PWH, early identification of their risk of death is crucial as it enables timely adjustments in follow-up methods and treatment regimens, ultimately enhancing their survival and quality of life. Unfortunately, effective tools for the early prediction of the risk of death are currently lacking. Therefore, discovering a more accurate method to predict the risk of death in PWH is of paramount importance. It not only improves the survival rate and quality of life for infected individuals but also optimizes the allocation of medical resources.

Traditional risk prediction methods primarily rely on clinical data and medical knowledge. However, with the advancements in big data and machine learning technology, employing machine learning algorithms to process and analyze extensive data has proven advantageous in disease diagnosis and prognosis prediction. Machine learning plays a crucial role in disease diagnosis by identifying individuals at high risk of developing the disease. This approach helps in screening out such individuals and allows for more targeted interventions. Traditional diagnostic methods for specific clinical diseases can be invasive or expensive, but with the integration of machine learning, we can enhance the accuracy of diagnosis for high-risk individuals. Additionally, machine learning methods enable us to predict disease prognosis, thereby helping to prevent or delay adverse outcomes effectively. By leveraging these techniques, we can significantly mitigate the impact of diseases. For instance, prognostic models have been established for predicting outcomes in chronic obstructive pulmonary disease patients [5]. Similarly, for cancer diagnosis, prognosis, and treatment [6]. In this context, some researchers have also endeavored to develop early predictive models for mortality in HIV-infected individuals. A meta-analysis systematic review was conducted in order to address the controversy surrounding the predictive value of diverse models for HIV-related death. The study aimed to identify an accurate, efficient, and widely applicable method for predicting death in HIV/AIDS patients. The findings of this review will provide decision-making support for clinicians and inform the development of improved treatment regimens for patients. Various studies have shown significant variations in the follow-up period, leading to the construction of different predictive models.

## Methods

### Study registration

Our study adhered to the systematic review and meta-analysis reporting guidelines (PRISMA 2020). Additionally, we proactively registered comprehensive details of the systematic review protocol on PROSPERO (ID: CRD42023488238).

### Eligibility criteria

#### Inclusion criteria

1. The included study subjects were diagnosed HIV-infected individuals;
2. The included study types were case-control studies, cohort studies, nested case-control studies, and case-cohort studies;
3. The complete construction of the death-related predictive model was achieved without restricting the follow-up time for death;
4. Some studies did not set up independent validation cohorts. However, we cannot ignore the collinearity of these studies in this field. During the meta-analysis process, we summarized the c-index of the training set and validation set to describe the existence of overfitting. Therefore, studies without independent validation sets were also included in our systematic review;
5. In some studies, different researchers may publish machine learning research based on the same dataset (especially authoritative registered databases). Due to the possibility of different modeling methods and modeling variables, those studies were also incorporated into our systematic review;
6. The included literature was reported in English in the research.

#### Exclusion criteria

1. Study types were meta-analysis, review, guideline, expert opinion, etc.
2. Only the analysis of risk factors or predictive factors for death in PWH was conducted, and no complete study of machine learning models was constructed;
3. The following outcome indicators for evaluating the accuracy of machine learning models were missing (ROC, c-statistic, c-index, sensitivity, specificity, accuracy, recovery rate, precision, confusion matrix, diagnostic fourfold table, F1 score, and calibration curve);
4. Studies with a small sample size (< 20 cases);
5. Studies on the univariate prediction accuracy;
6. Conference abstracts published without peer review.

### Data sources and search strategy

During our systematic exploration, we meticulously combed through the PubMed, Cochrane, Embase, and Web of Science databases, with the search cutoff date configured to May 26, 2023. To mitigate the potential of overlooking recently published literature, we additionally performed searches on November 25, 2023, within the aforementioned databases. The search was executed employing both subject terms and free-text terms, devoid of any constraints on region or publication year. Comprehensive search strategies are delineated in Additional Material 1.

### Study selection and data extraction

We imported the retrieved literature into EndNote and employed a combination of automated and manual methods to identify duplicate publications. Following this, we thoroughly reviewed the titles and abstracts to preliminarily screen the original studies that met the criteria. Subsequently, we downloaded the full texts of these studies. The original studies that ultimately fulfilled the criteria for our systematic review underwent further screening based on their full texts. Before proceeding with data extraction, we established a standardized data extraction spreadsheet. This spreadsheet included the following categories: Title, First author, Years of publication, Author country, Study type, Patient source, Follow-up duration, Cause of death, Number of deaths, Total number of cases, Number of death cases in the training set, Total number of cases in the training set, Generation mode of the validation set, Overfitting methods, Verification of the number of deaths in the set, Number of cases in the validation set, Missing value processing method, Variable screening/feature selection methods, Use of model types, and Modeling variables.

The literature screening and data extraction mentioned above were independently conducted by two researchers (LYF, HXY). After completion, a cross-check was performed. In the event of any disputes, resolution will be sought through consultation with the third researcher (NMJ).

### Risk of bias in studies

We utilized PROBAST to evaluate the bias risk of the original study, encompassing a comprehensive set of questions across four distinct domains: participants, predictive variables, results, and statistical analysis. These domains comprised 2, 3, 6, and 9 specific questions, respectively, each having three response options (yes/possibly yes, no/possibly no, and no available information). If any answer in a domain indicated “no” or “possibly no,” it was deemed high risk. Conversely, for a domain to be considered low risk, all questions needed “yes” or “possibly yes” responses. The overall bias risk was determined as low when all domains were classified as low risk. Conversely, if at least one domain was designated as high risk, the overall bias risk was deemed high. Bias risk assessments were independently conducted by two researchers (LYF, HXY) using PROBAST, with cross-verification upon completion. In the event of disagreements, a third researcher (NMJ) was consulted for resolution.

### Outcomes

The primary outcome indicator in our systematic review was the C-index, reflecting the overall accuracy of the predictive model. The review focused on assessing the risk of death in HIV-infected individuals and identified variations in different follow-up times. Some original studies developed survival analysis models, such as COX regression, Fine & Gray model, random survival forest, etc. The performance of these models, as indicated by the area under the ROC curve, varied over time, emphasizing the need for the C-index to describe their effectiveness. In contrast, non-survival analysis models, including logistic regression, random forest, and support vector machine, produced outcome indicators with a consistent area under the ROC curve that did not vary with time. These models demonstrated performance equivalent to the C-index observed in survival analysis models.

### Synthesis methods

We conducted a meta-analysis of the c-index, an indicator used to assess the overall accuracy of machine learning models. In cases where the 95% confidence interval and standard error of the c-index were not provided in original studies, we referred to the work of Debray TP et al. (Debray TP, Damen JA, Riley RD, et al. A framework for meta-analysis of prediction model studies with binary and time-to-event outcomes. Stat Methods Med Res 2019;28:2768-86.) to estimate its standard error. Considering variations in variables and parameter inconsistencies across different machine learning models, we prioritized the use of random effects models for the meta-analysis of c-index.

In addition, we employed a bivariate mixed-effects model for a comprehensive meta-analysis of sensitivity and specificity. During the meta-analysis process, sensitivity and specificity values were derived from the diagnostic fourfold table. However, a significant number of original studies did not provide this table. In such instances, we utilized two approaches to calculate the diagnostic fourfold table: (1) Computation based on sensitivity, specificity, precision, and the number of cases; (2) Extraction of sensitivity and specificity using the optimal Youden’s index, followed by calculation with the number of cases. The meta-analysis for this study was performed using R 4.2.0 (R Development Core Team, Vienna, http://www.R-project.org).

## Results

### Study selection

**Fig. 1:**
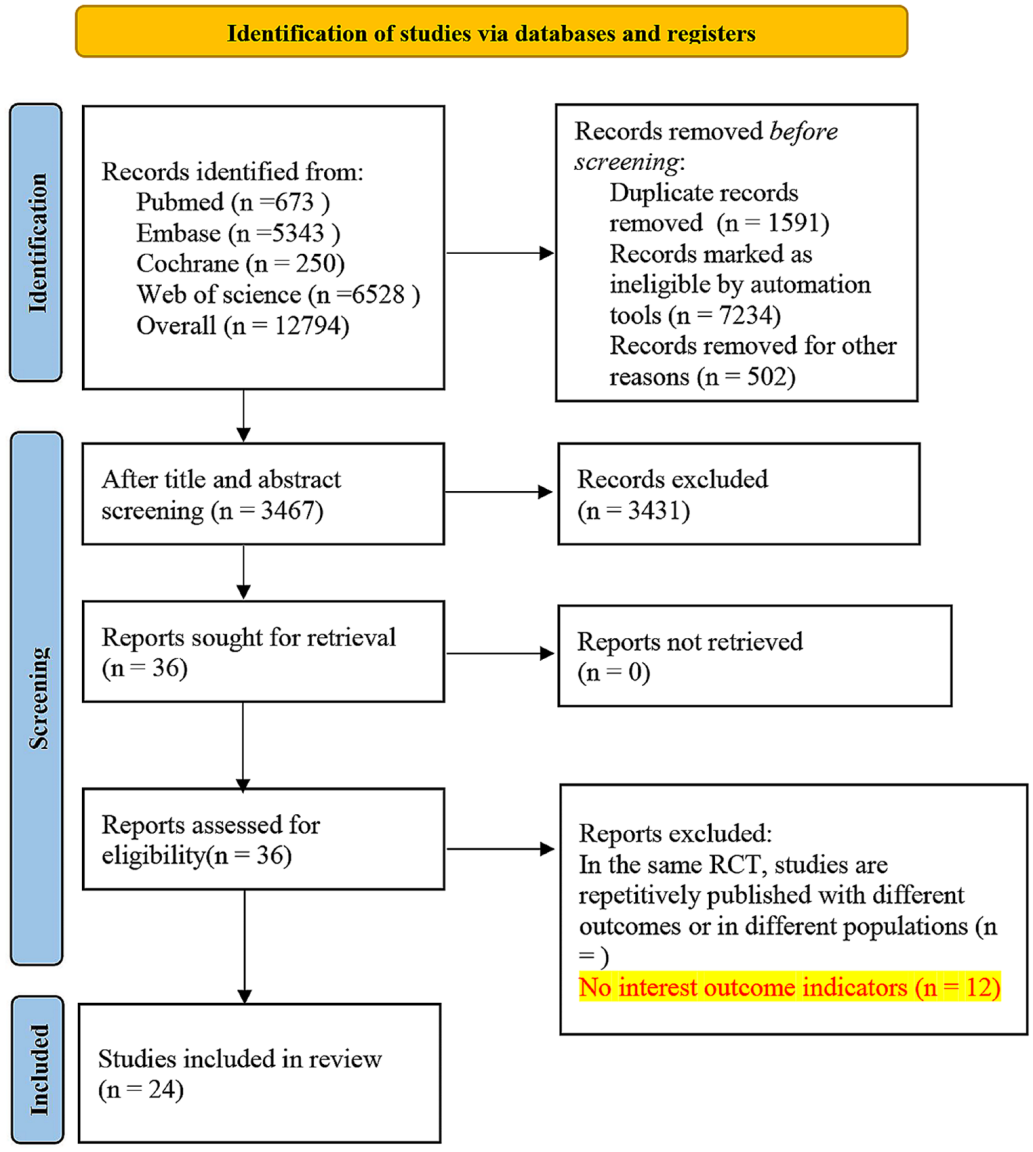
Literature screening process

We conducted a comprehensive search across PubMed, Cochrane, Embase, and Web of Science databases, identifying a total of 12,794 pieces of literature. Out of these, 1,591 were identified as duplicate articles and subsequently removed. Following the elimination of duplicates, we performed initial screening based on titles and abstracts, ultimately pinpointing 36 articles relevant to our research topic. Upon downloading and thoroughly reviewing the full texts of these articles, we excluded the following categories: 2 articles lacking detailed classification of HIV-infected individuals and their deaths, 3 studies concentrating on methodological modeling improvements or economic evaluation indicators without patient data, 3 pieces of literature featuring outcome indicators inconsistent with our research focus, and 4 studies utilizing bioinformatics methods to assess the risk of death in HIV-infected individuals at the individual level. Ultimately, our refined selection includes a total of 24 previous studies [7–30] that align with our research topic. The specific screening process is visualized in Fig. 1.

### Study characteristics

We reviewed 24 studies(Table 1), encompassing a total of 401,389 individuals living with HIV. These studies were conducted in various countries and regions, including 7 from the United States [15, 18, 19, 21, 24, 25, 28], 10 from China [7–11, 13, 14, 16, 20, 23], and the rest from Spain [12], Mozambique [17], Germany [22], Congo [26], Uganda [27], Switzerland [29], and Canada [30].

**Table 1:** Study characteristics

No.	First author	Year of publication	Author’s nationality	Types of study grouping	Patient source	Followtime(year)	Classification of causes of death	Number of death cases	Total number of cases	The total number of cases in the training set	Method of generating the validation set	Number of cases in the validation set	Types of models used	Modeling variables
1	Haili Wang	2023	China	Cohort studies	Database	36	Kaposi’s sarcoma death	12,943	17,103				Fine and Gray model, nomogram	sex, age at diagnosis, marital status at diagnosis, race, current smoking prevalence, ever-smoking prevalence, registries, countylevel socioeconomic status (SES), year of diagnosis, tumour extent of KS, follow-up time, survival outcomes, and cause of death
2	Minjuan Shi	2022	China	Cohort studies	Single-center	In-hospital death	In-hospital death	256	1927	1348	Random sampling (7:3)	579	logistic regression, eXtreme Gradient Boosting (XGBoost), K-nearest neighbors (KNN), and support vector machine (SVM)	CD3, CD3 + T-cell count; CD4/CD8, CD4/CD8 ratio; CD8 + T-cell count; LDL, low-density lipoprotein cholesterol; Ca, calcium; HDL, high-density lipoprotein cholesterol; CREA, creatinine; AST, aspartate aminotransferase; UA, uric acid; LDH, lactate dehydrogenase; Ccr, endogenous creatinine clearance rate; Glu, glucose; CHOL, total cholesterol; TBIL, total bilirubin; AST/ALT, AST/ALT ratio; BUN/CREA, BUN/CREA ratio; BUN, urea nitrogen; K, potassium; IBIL, indirect bilirubin; P, phosphorus; Cl, chlorine; Na, sodium; STY, osmolarity; HCO3, carbonate; Cys-C, serum cystatin C; AG, anion gap; DBIL, direct bilirubin; TBA, total bile acid; Hb, hemoglobin; PLT, platelet; MONO%, monocyte ratio; RDW-CV, red blood cell distribution width; HCT, hematocrit; EOS, eosinophil; EOS%, eosinophil ratio; PDW, platelet distribution width; PCT, platelet distributing width; CRP, C-reactive protein; hsCRP, high-sensitivity C.
3	Sara Domı´nguez-Rodrı´guez	2022	Spain	Cohort studies	multicenter	1	OS	22	100	100	Random sampling (7:3)	280	LR, Random Forest (RF), Support Vector Machine (SVM), K-Nearest Neighbor (KNN), Naïve Bayes (NB), Artificial Neural Network (ANN), and Elastic Net	sociodemographic, virologic, immunologic, and maternal status features
4	Xueqin Li	2022	China	Cohort studies	multicenter	8	OS		121				**Cox**	(26 variables) In univariate analysis, factors associated with poor OS include CD4 count of 100 cells/mL, mediastinal or hilar lymph nodes, liver involvement, gastrointestinal tract involvement, extracapsular invasion, necrosis within the lesion, and treatment without chemotherapy [Table 2]. Multivariable Cox analysis showed that involvement of mediastinal or hilar lymph nodes, liver, necrosis of the lesion, CD4 count of 100 cells/mL, and treatment without chemotherapy were independent risk factors for short OS.
5	Fangfang Jiang	2022	China	Nested case-control studies	Single-center	17	OS	120	600	420	bootstrap	180	COX	CD4, BMI and HB were preliminarily selected to construct a prediction model of three-year and five-year survival of PLHIV after ART.
6	Qiuyue Feng	2022	China	Cohort studies	multicenter	18	Pneumocystis pneumonia death	84	360				Nomogram	28 univariate prognosis analyses, 10 multivariate death regression analyses
7	Ying Chen	2022	China		Registered database	10	OS				External validation	The incidence and mortality from 2014 to 2019 served as the test set.	ARIMA and LSTM hybrid model	
8	Ting Zhao	2021	China	Cohort studies	multicenter	8	OS	81	903	386	External validation	169	risk-scoring model	Disturbance of consciousness, stiff neck, age, TBIL, ICP, PLT levels, HIV RNA viral load, cerebrospinal fluid glucose, and urea levels were significantly correlated with patient mortality rate (*p* < 0.05).
9	Chang Shu	2021	USA	Cohort studies	Single-center	10	OS	276	1081	460	Random sampling	114	Cox, RF, GLMNET, SVM, and k-NN	Clinical information on patient age, gender, race, smoking status, CD4 count, viral load, HIV medication compliance, VACS index, and mortality rate.
10	Yuanyuan Qin	2021	China	Cohort studies	multicenter	10		25	384	384	Multicenter external validation	233	logistic regression model.	Demographic statistics, comorbidities, symptoms, signs and results of laboratory tests when admitted, including levels of hemoglobin, platelet count, white blood cell count, aspartate aminotransferase/alanine aminotransferase (AST/ALT) ratio, albumin level, total bilirubin level, creatinine level, lactate dehydrogenase (LDH), CD4 + T cell count, and blood urea nitrogen (BUN) level
11	Laura Fuente-Soro	2021	Mozambique	Cohort studies	multicenter	4	OS	149	3486				Fine & Gray model (FGR)	
12	Yanink Caro-Vega	2021	USA	Cohort studies	Database	2	OS	102	1823				Fine & Gray model (FGR)	7 variables
13	Z. Yuan	2020	China	Cohort studies	Single-center	0	OS	485	5711	3724	External validation	1987	Cox	Occupation, antiretroviral treatment (ART), pneumonia, tuberculosis, Talaromyces marneffei, hypertension, sepsis, anemia, respiratory failure, hypoproteinemia, and electrolyte imbalance.
14	Gina Turrini	2020	USA	Cohort studies	multicenter	5	OS	1435	6927				Cox, logistic regression model, Fine & Gray model (FGR)	Gender, race, and ethnicity (non-Hispanic blacks, non-Hispanic whites, non-Hispanic Asians, and Hispanics), dual enrollment in medical subsidy and insurance (representing low-income status), residence in rural (or non-core statistical areas [46]) or urban areas, and their indicator variables at age 65 (time changes in controlling factors such as treatment and cohort composition). Depression, hypertension, chronic kidney disease, chronic obstructive pulmonary disease (COPD), osteoporosis, heart disease, colorectal cancer, lung cancer, diabetes, chronic hepatitis and end-stage liver disease.
15	Nico Reinsch	2019	Germany	Cohort studies	multicenter	10	OS	158	808				Cox	Age, gender, Framingham risk, diabetes, hypertension, cardiovascular events, number of cardiovascular events
16	Xiangqing Hou	2019	China	Nested case-control studies	Single-center	12	OS	150	750	525	Random sampling	225	Cox	CD4, VL, HB, GLU, and CR
17	Harshith R. Avula	2019	USA	Cohort studies	multicenter	10	OS	36	448				Cox	KP location and calendar era, population characteristics (age, gender, and self-reported race), community-level socioeconomic status (income level and education level), lifestyle factors (smoking status, alcohol abuse, and illegal drug use), baseline medical history (i.e. myocardial infarction, unstable angina, coronary artery bypass surgery, percutaneous coronary intervention, atrial fibrillation/flutter, valvular heart disease, peripheral artery disease), ischemic stroke or transient ischemic attack, diabetes, hypertension, dyslipidemia, depression, dementia, chronic liver disease, chronic lung disease, hypothyroidism, hyperthyroidism, hospitalization for bleeding, cancer, and receipt of cardiac implantable electronic devices), outpatient vital signs (systolic blood pressure and BMI), renal function (estimated glomerular filtration rate and proteinuria), LVEF category, and use of targeted drugs (i.e. angiotensin-converting-enzyme inhibitors/angiotensin II receptor blockers, beta-blockers, calcium channel blockers, diuretics, alpha-antagonists, aldosterone receptor blockers, statins, non-statin lipid-lowering agents, anticoagulants, antiplatelet agents, diabetes medications, and non-steroidal anti-inflammatory drugs).
18	Ruibin WANG	2018	USA	Cohort studies	multicenter	10	OS	74	841				Cox	
19	Margaret L. McNairy	2018	USA	Cohort studies	multicenter	2	OS	242	7031	7031	External validation	1835	logistic regression models	Age, gender, weight, CD4 count, WHO staging, and tuberculosis (TB) diagnosis
20	James Nugent	2014	Democratic Republic of Congo	Cohort studies	multicenter	8	OS	63	1010	673	Random sampling	337	Cox	Age, gender, symptoms or status of HIV, WHO clinical staging, CD4 ratio, CD4 count, history of tuberculosis, hemoglobin, age-specific weight Z-score, height-specific age Z-score, and total lymphocyte count
21	Agnes N. Kiragga	2013	Uganda	Cohort studies	Single-center	3	OS			5633		559	Cox	Age, gender, WHO clinical stage, baseline ART regimen)
22	Todd H. Driver	2013	USA	Cohort studies	multicenter	10	OS		1197				Cox, Bayesian model	Traditional risk factors for population characteristics, CVD and CKD, and HIV-specific clinical factors
23	Matthias Egger	2011	Switzerland	Cohort studies	multicenter	2	OS	1363	24,257		Monte Carlo simulations		Cox	
24	G.E. Hatzakis	2002	Canada	Cohort studies	Single-center	18	OS	94	116				Cox, ANN	Virus load, CD3%, CD4%, CD8%, CD3 absolute value, CD4 absolute value, and CD8 absolute value levels

These studies comprised 22 cohort studies [31–52]and 2 nested case-control studies [7–30]. In terms of patient sources, 15 studies originated from multicenter sources [31, 33–35, 37, 39, 40, 42, 44–48, 50, 51, 53], 2 were drawn from a registered database [13, 18], and 7 were conducted at single centers [8, 10, 15, 20, 23, 27, 30]. In a follow-up report, one study specifically focused on in-hospital deaths resulting from the combination of Talaromyces marneffei and HIV infection [8], while the remaining studies reported deaths during long-term follow-up, with the longest follow-up period extending to 36 years [7]. The majority of studies concentrated on all-cause mortality in HIV-infected individuals, with only two studies reporting deaths attributed to Kaposi’s sarcoma [7] and Pneumocystis jirovecii Pneumonia [11]. In the training set, a total of 14,148 cases of deceased individuals were recorded, encompassing 3 types of models. The generation of the validation set involved internal random sampling and external validation, with external validation utilizing two modes: prospective and multicenter. The 8 studies employed survival analysis models (COX regression) [7, 9–11, 20, 23, 26, 30], while the remaining 16 studies utilized non-survival analysis models [32, 35–42, 44–46, 49–51]. The modeling variables are detailed in the Additional Materials.

### Risk of bias in studies

**Fig. 2:**
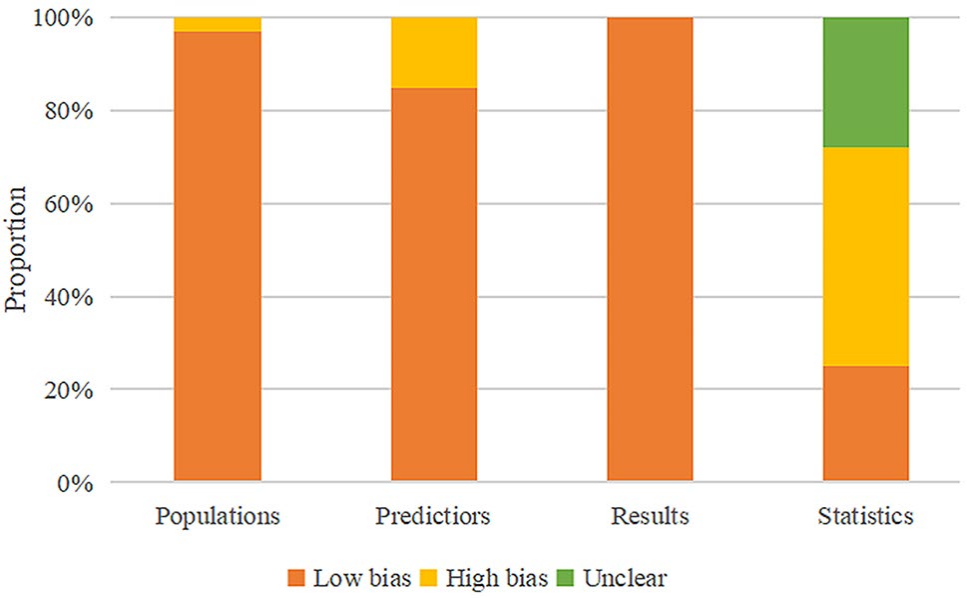
PROBAST assessment of the original study for quantitative analysis

The assessment of the original studies utilized the PROBAST evaluation tool. Regarding the study subjects, an article with data sourced from retrospective cohort studies [11] is considered to have a high bias. Additionally, an article studying in-hospital mortality among infected individuals makes it challenging to assess predictive factors without knowing the outcomes, resulting in high bias [8]. In the evaluation of results, due to the particularity of the outcome indicator being death, the evaluation results related to the definition of the outcome in the included articles are all low in bias. In statistical analysis, most non-survival analysis studies meet the criterion of EPV ≥ 20, and a sample size of an independent validation set ≥ 100 indicates low bias. However, survival analysis studies using COX regression and the Fine & Gray model (FGR) do not establish independent external validation [9, 13, 18, 21, 22, 28, 29]. In some studies, the rarity of cases makes it challenging to meet the conditions of EPV > 20 or an independent validation sample size > 100, leading to high bias [7, 10–12, 14, 16, 17, 24, 26] (Fig. 2).

### Meta-analysis

#### Training set

##### Synthesized results

**Fig. 3:**
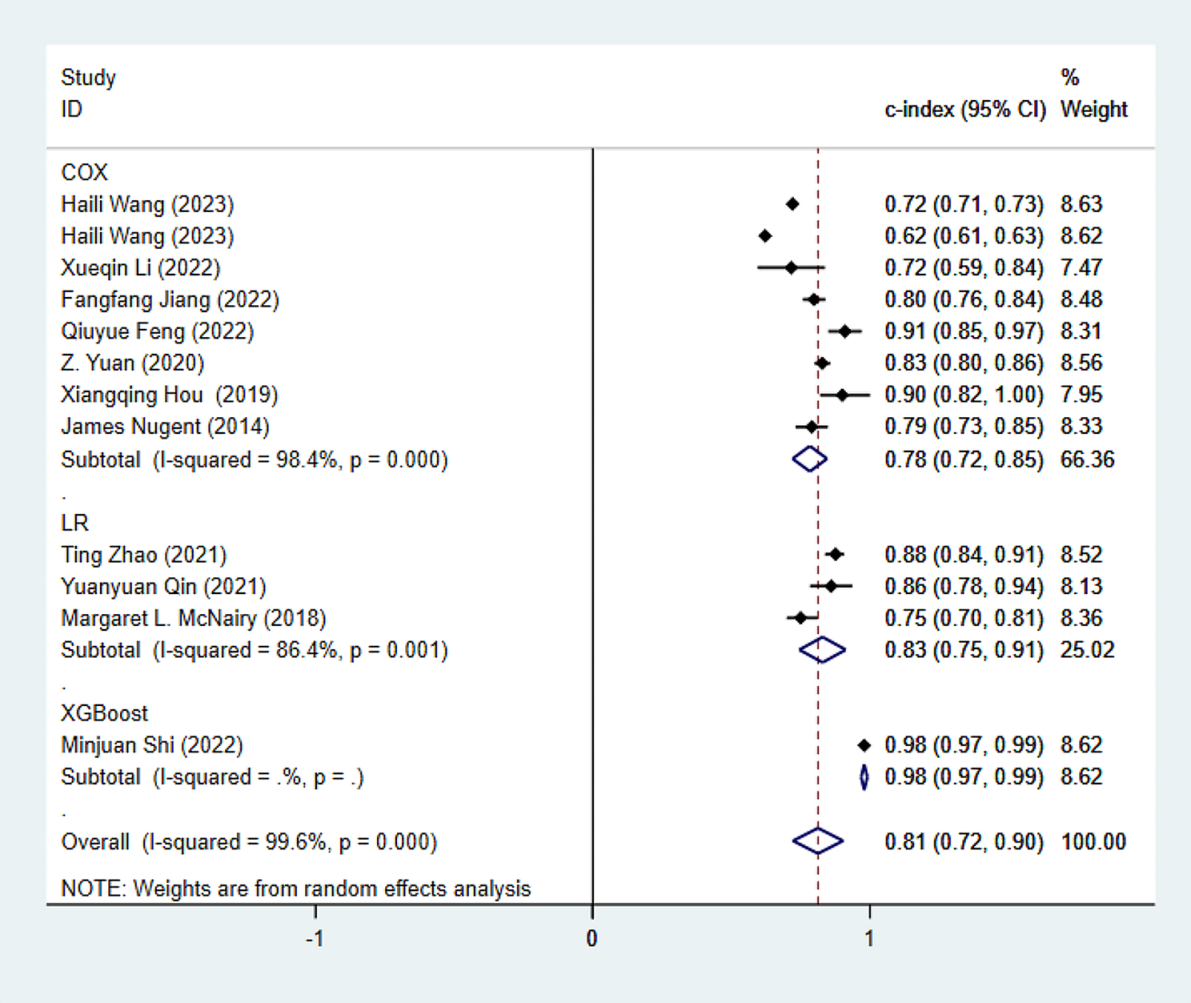
Forest plot of the c-index meta-analysis of predictive models for PWH death prediction in the training set

Within the training set, there are a total of 12 models, and the c-index obtained through the aggregation of random effects models is 0.81 (95% CI: 0.72–0.90). The summarized c-index for the LR model is 0.83 (95% CI: 0.75–0.91), while the summarized c-index for the Cox model is 0.78 (95% CI: 0.72–0.85) (Fig. 3).

##### Sensitivity analysis and reporting biases

**Fig. 4:**
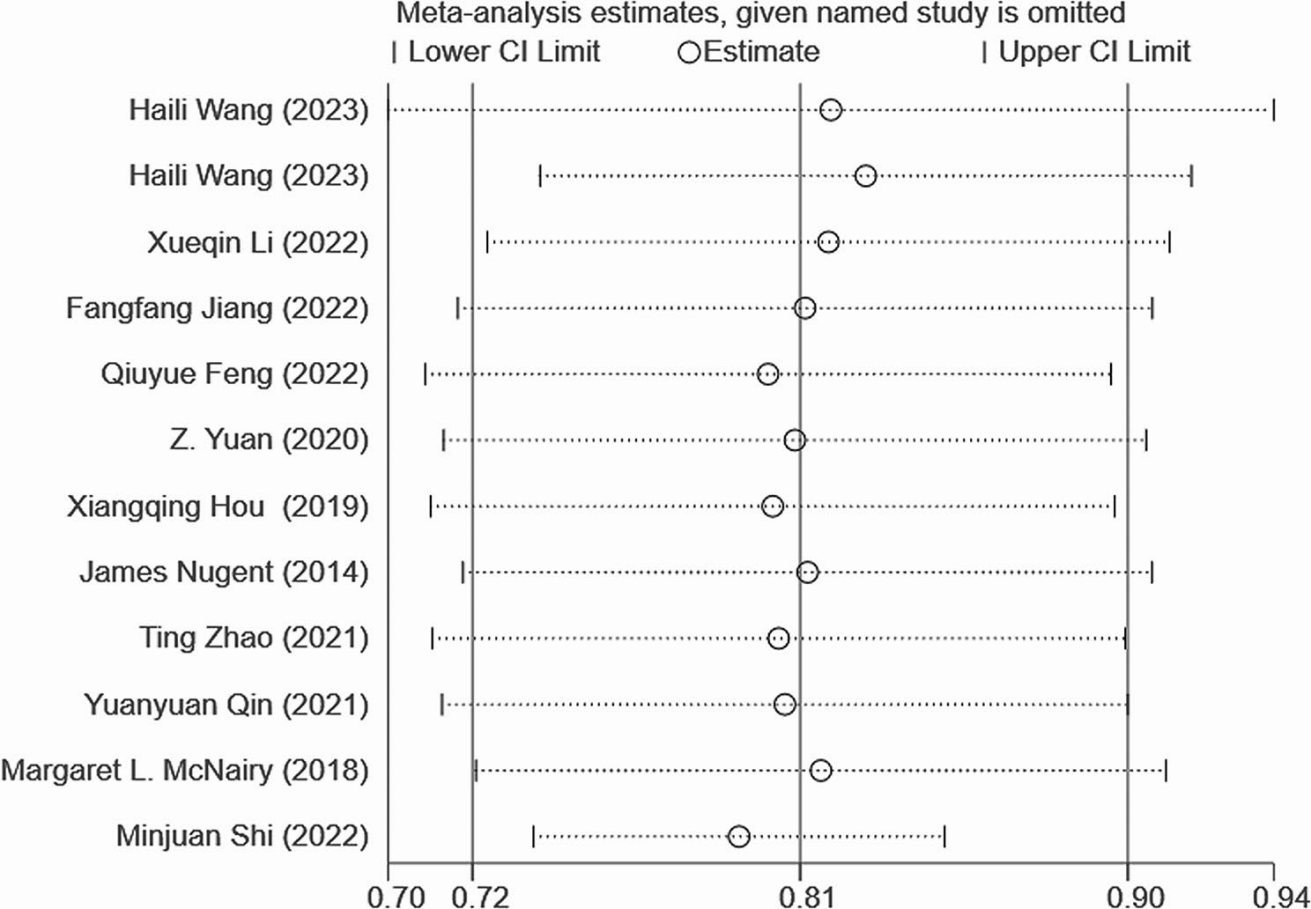
Forest plot of the sensitivity analysis of the c-index meta-analysis of the predictive models for PWH death in the training set

**Fig. 5:**
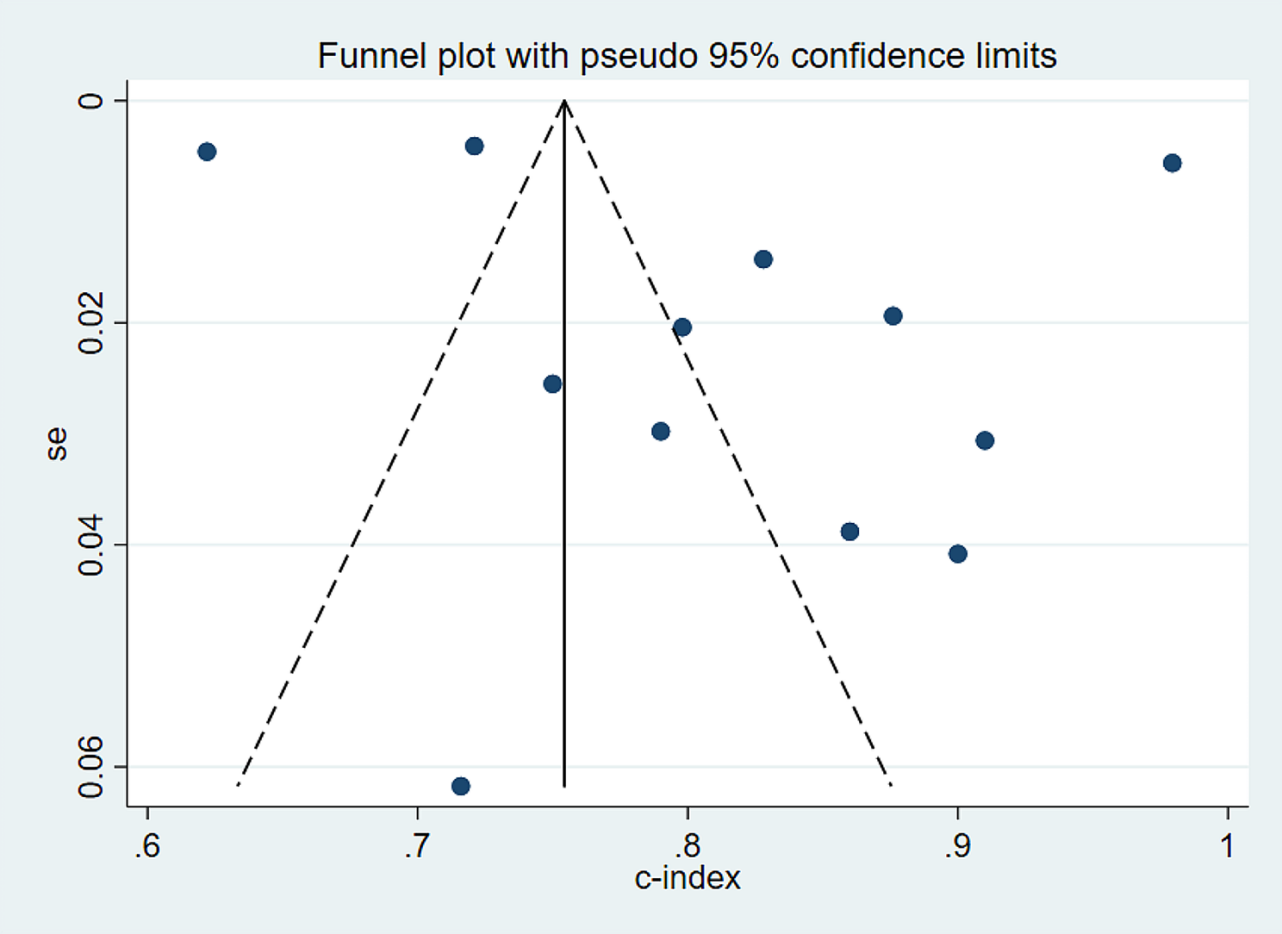
Funnel plot of the c-index meta-analysis of the predictive models for PWH death in the training set

During the sensitivity analysis of the training set in this study, we systematically excluded each model and summarized the results of the remaining ones. The findings suggest that even after removing each model, the results remain stable (Fig. 4). Additionally, the funnel plot reveals no evidence of publication bias, and the Egger test yields a p-value of 0.468 (Fig. 5).

##### Meta-regression

**Fig. 6:**
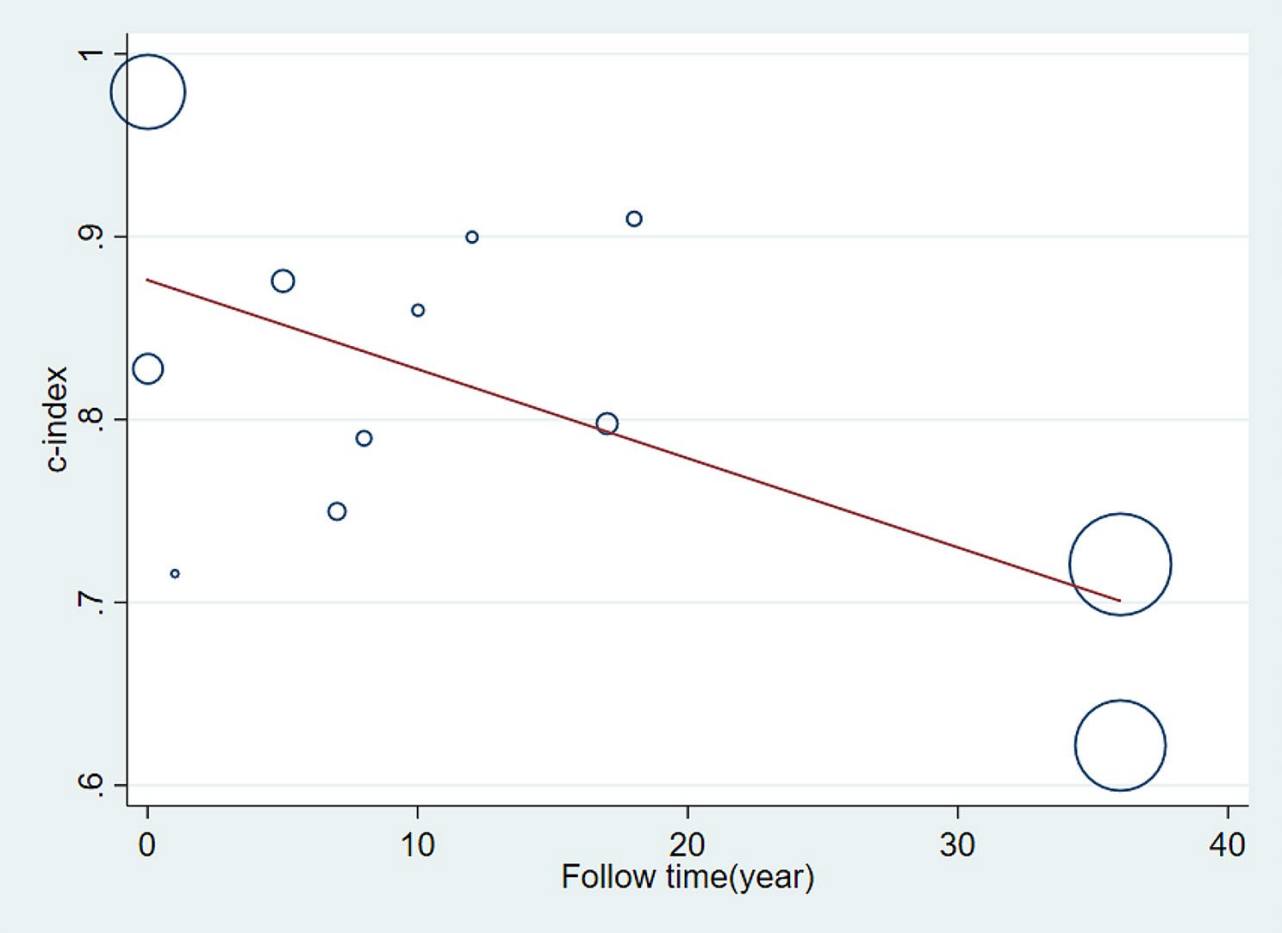
Meta-regression analysis of the follow-up time for death prediction of PWH by the predictive model in the training set

Meta-regression analysis was conducted on the follow-up time of the training set in these studies. The adjusted R2 reveals that 38.40% of the inter-study variance has been explained. Following Knapp-Hartung adjustment, the coefficient for follow-up time is -0.0048738, with a standard error of 0.0019694. The t-value is -2.47, and the p-value is 0.033 (*p* < 0.05), indicating a significant impact of varying follow-up times on the c-index. With increasing follow-up time, there is a noticeable declining trend in the c-index, as illustrated in Fig. 6.

#### Validation set

##### Synthesized results

**Fig. 7:**
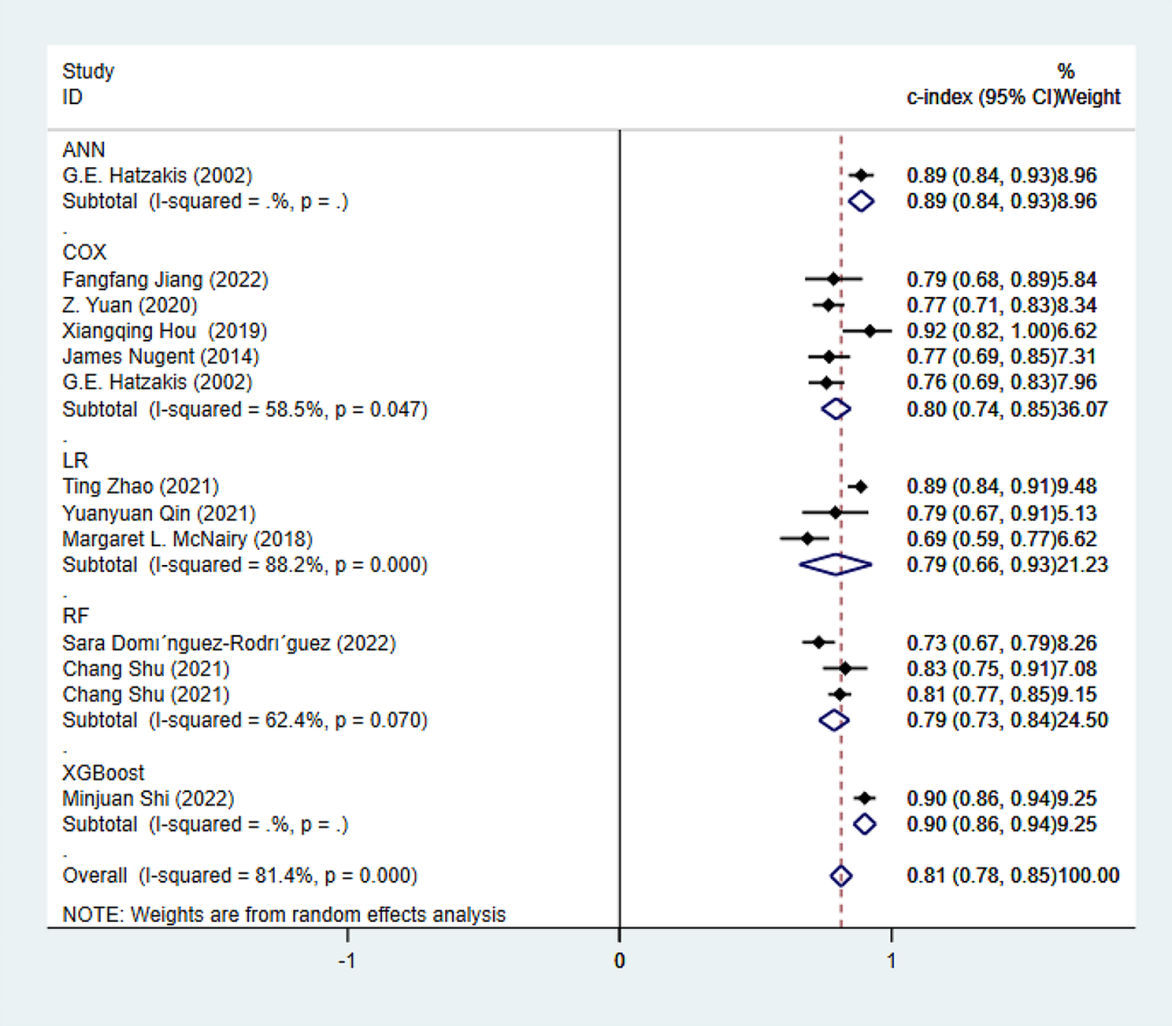
Forest plot of c-index meta-analysis of the prediction for PWH death by predictive models in the validation set

In the validation set, 13 models were included, and the c-index, summarized using random effects models, was 0.81 (95% CI: 0.78–0.85). Specifically, the summarized c-index for LR is 0.79 (95% CI: 0.66–0.93), and for Cox, it is 0.80 (95% CI: 0.74–0.85) (Fig. 7).

##### Sensitivity analysis and reporting biases

**Fig. 8:**
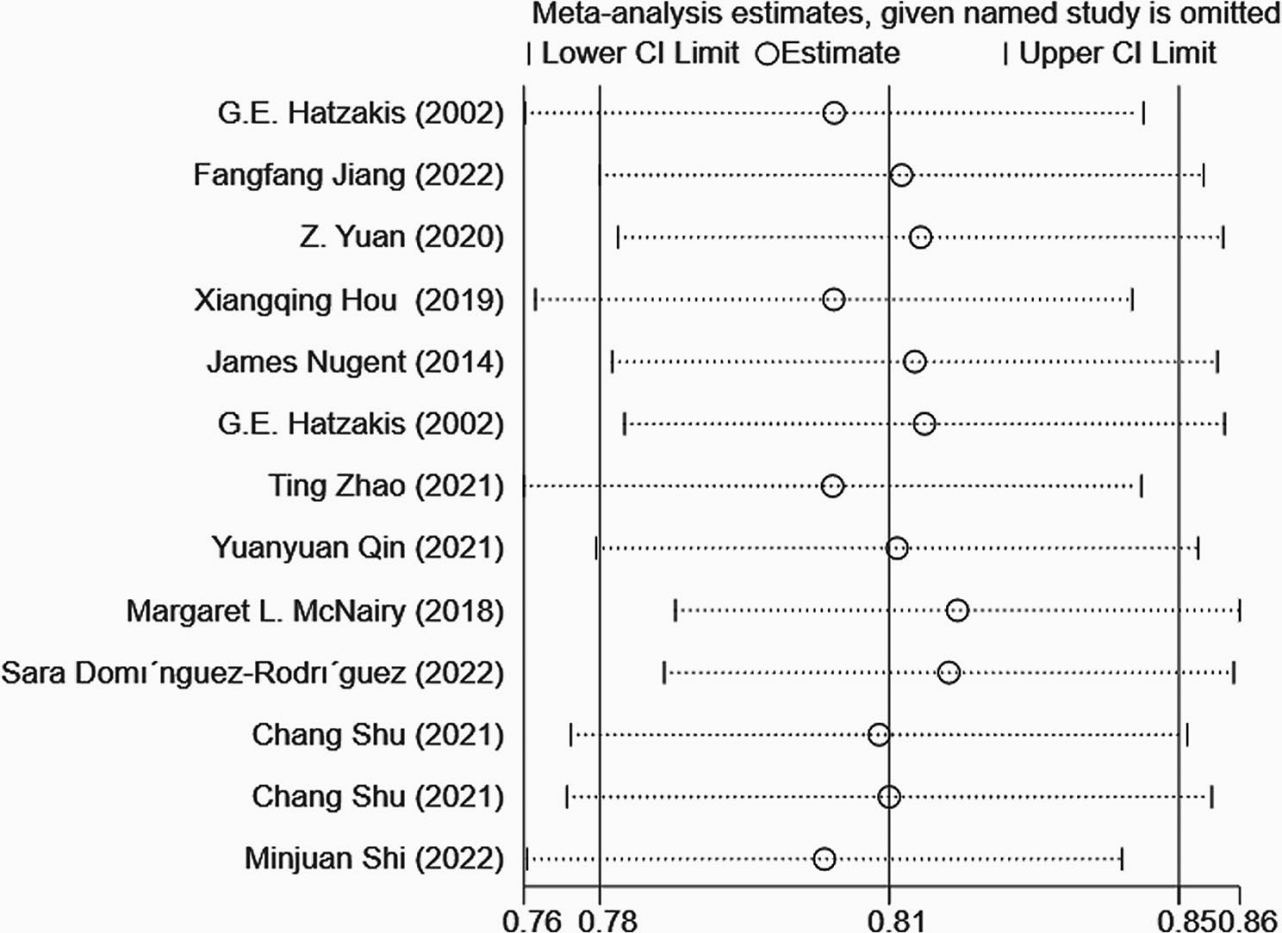
Forest plot of the sensitivity analysis of the c-index meta-analysis of the predictive models for PWH death in the validation set

**Fig. 9:**
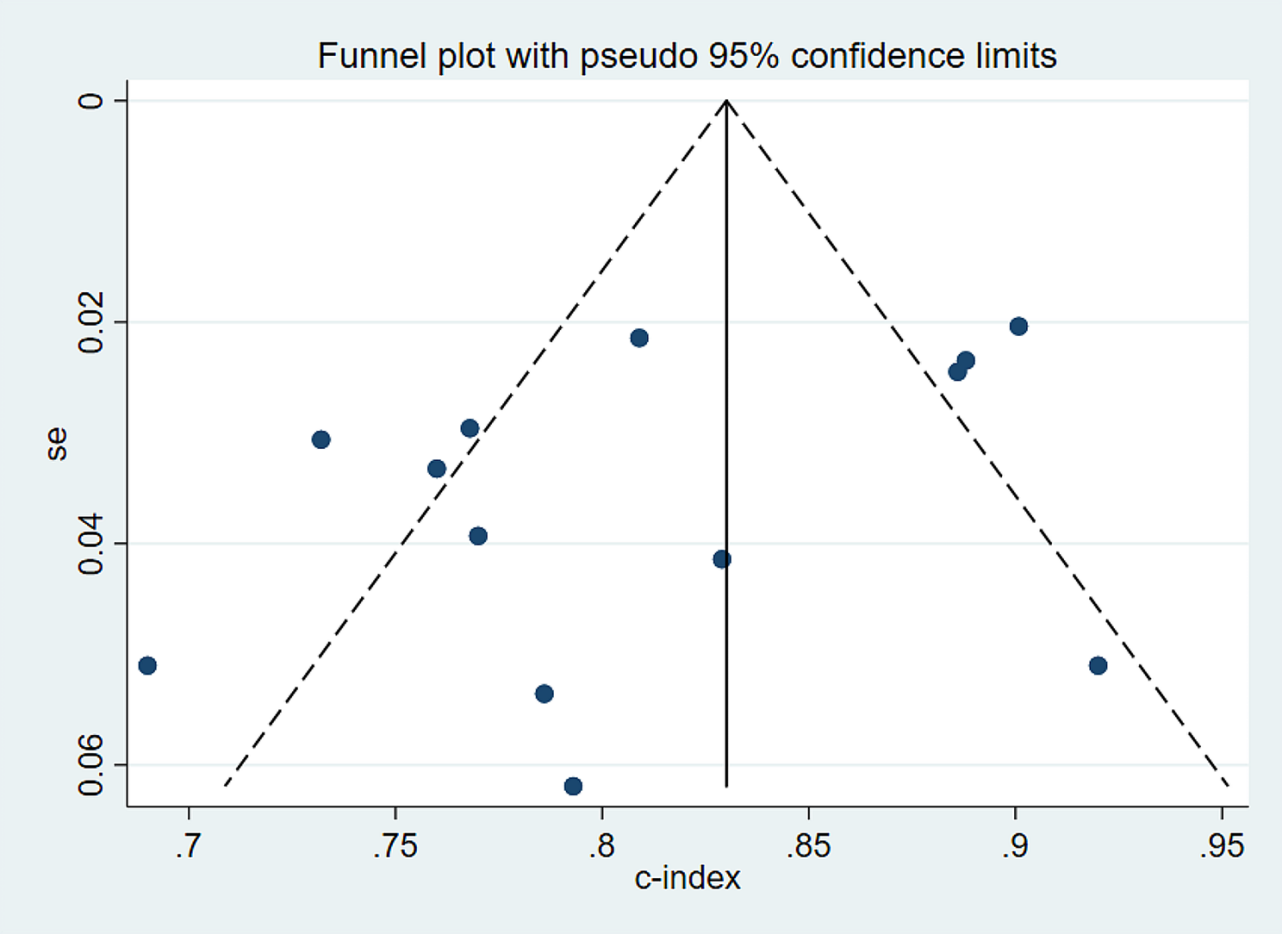
Funnel plot of the c-index meta-analysis of the predictive models for PWH death in the validation set

The sensitivity analysis results for the validation set indicate that the summarized findings remain consistent even after systematically excluding models one by one (Fig. 8). Furthermore, the funnel plot did not indicate any publication bias, with Egger’s test showing a p-value of 0.118 (Fig. 9).

##### Meta-regression

**Fig. 10:**
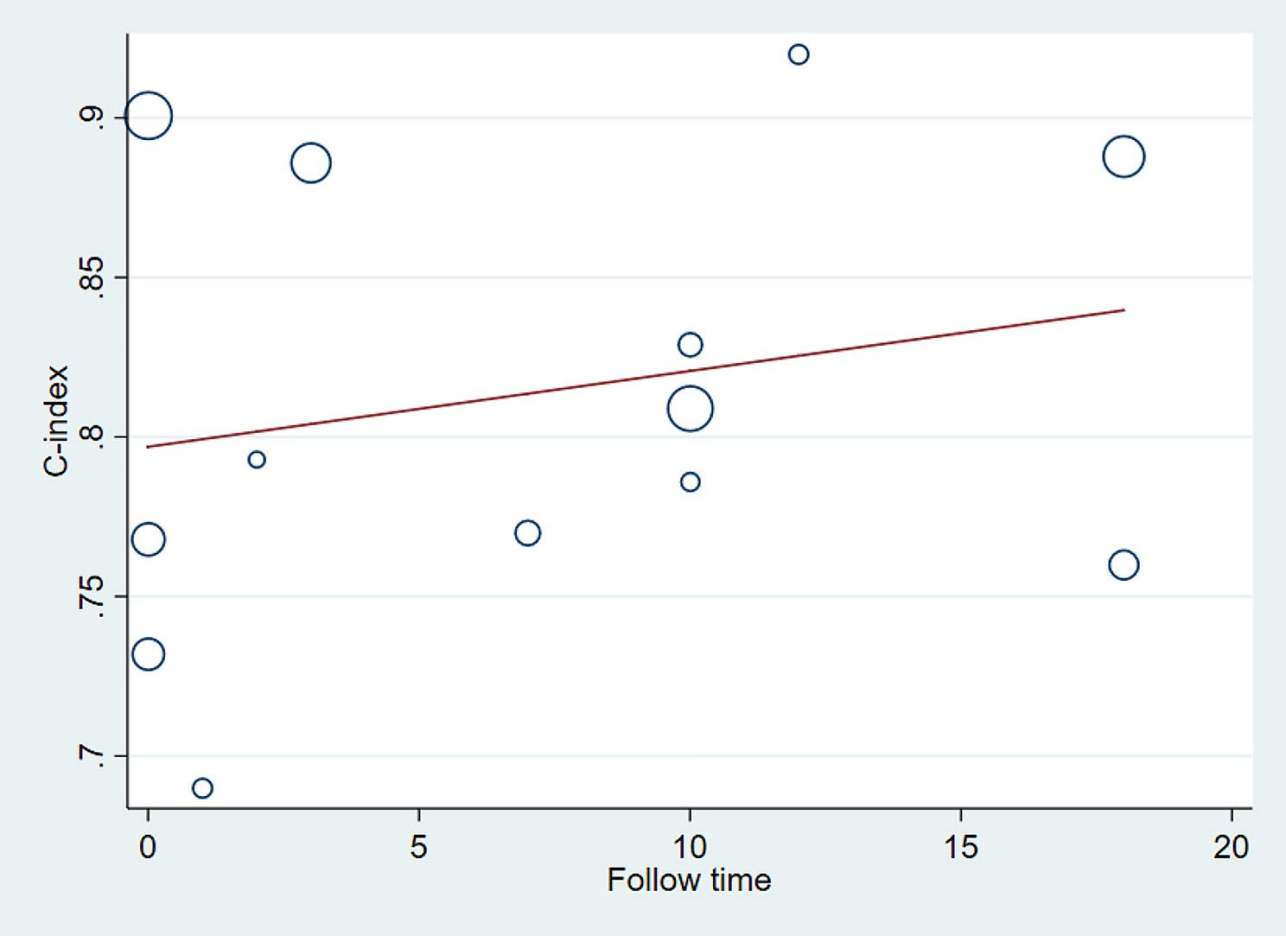
Meta-regression analysis of follow-up time of predictive models for PWH death in the validation set

After conducting meta-regression analysis on the follow-up time of the validation set, the results are as follows: The REML estimated inter-study variance is 0.003958, and 80.73% of the residual variation is attributed to heterogeneity. The adjusted R-squared is -8.29%. Following Knapp-Hartung adjustment, the intercept term is 0.7968903 with a standard error of 0.0298499. The coefficient for follow-up time is 0.0023765 with a standard error of 0.0031152. The t-value is 0.76, and the p-value is 0.462 (*p* > 0.05), indicating that the effect of follow-up time on the c-index is not significant. (Fig. 10).

## Discussion

### Summary of the main findings

The objective of this comprehensive systematic review and meta-analysis is to assess the efficacy of machine learning models in predicting the risk of death among HIV/AIDS patients. Following a meticulous database search and utilizing the Prediction Model Risk of Bias Assessment Tool (PROBAST) for bias risk evaluation, we identified 24 eligible studies encompassing 401,389 People with HIV (PWH). These studies predominantly center on the mortality of outpatients during extended follow-up periods and have employed various machine learning models, encompassing both survival and non-survival models. The meta-analysis reveals that machine learning models exhibit robust performance in predicting the risk of death among PWH, with a c-index of 0.83 (95% CI: 0.75–0.91) in the training set and a c-index of 0.81 (95% CI: 0.78–0.85) in the validation set. Furthermore, meta-regression analysis indicates that the length of follow-up time does not significantly impact the performance of machine learning models in predicting PWH mortality. Demonstrating excellent predictive capabilities, the machine learning model performs admirably in forecasting the risk of death for PWH, as evidenced by the high c-index values in both the validation and training sets. This underscores their potential utility in clinical practice. These findings underscore the accuracy and reliability of machine learning models in aiding healthcare professionals to identify high-risk patients and optimize intervention strategies, ultimately improving patient prognosis.

### Comparison with previous reviews

In the realm of artificial intelligence, the application of AI to HIV has garnered widespread attention from researchers. In earlier studies, scholars James Stannah and Luo Qianqian conducted a meta-analysis of HIV infection risk among men who have sex with men (MSM) in high-risk populations. They employed Bayesian generalized linear mixed-effect models and meta-regression analysis to scrutinize trends in HIV testing, treatment cascade, and HIV incidence among MSM in Africa [54]. Another study synthesized 18 evaluation models, revealing that machine learning models exhibit fair to good discriminatory performance in predicting HIV infection risk (AUC 0.62, 95% CI: 0.51 to 0.73) [55]. Machine learning also demonstrates promising predictive and evaluative effects in clinical antiretroviral treatment (ART) [56] and pre-exposure prophylaxis (PrEP). For instance, Bayesian network meta-analysis (NMA) summarization disclosed that at week 96, there is improved differentiation in the efficacy, safety, and durability of dolutegravir when taken prior to exposure [57]. Furthermore, in recent years, some scholars have delved deeper into analyzing the treatment and immune changes of HIV-infected individuals with concurrent infections (tuberculosis [58], COVID-19 [59]) using multiple machine learning models. The application of vaccine-induced immune factors [60] has also found relevance in this domain. In order to enhance our understanding of survival status in individuals living with HIV, it is crucial to continue the discourse on this topic, despite the previous meta-analyses conducted. Hence, we conducted an assessment of the efficacy of machine learning models in predicting the risk of death among People living With HIV (PWH). Our objective was to complement earlier research findings and investigate the potential of machine learning in predicting early death risk among HIV/AIDS patients. By doing so, we aim to provide evidence-based suggestions for the advancement and refinement of intelligent prediction tools in this field.

Machine learning relies on modeling variables as key factors for enhancing accuracy. In the incorporated models, factors predicting death encompass common demographic characteristics, CD4 cell count, and viral load (VL), along with behavioral, biochemical, and antiviral therapy-related factors. Additionally, predictive factors, such as comorbid infection-related elements, primarily focus on observing the latency period of the disease course in HIV-infected patients. Monitoring these predictive factors during subsequent disease progression, particularly during the onset of AIDS, is crucial. Real-time monitoring or updating of these predictive factors will contribute to a more precise prediction of the risk of death. Therefore, vigilance towards changes in these predictive factors and timely adjustments to the model can significantly enhance prediction accuracy.

Other researchers have conducted similar systematic reviews regarding the prediction of death/positive events at different time points. For instance, Jin Jin examined the use of machine learning to predict the postoperative recurrence of hepatocellular carcinoma resection [61]. The study found that the model’s prediction method yielded favorable results, particularly when there were significant time differences. Additionally, studies have explored the prediction of disease-free survival (DFS) in breast cancer [62], as well as the assessment of chronic kidney disease risk and patient prognosis [63]. In this particular study, we examined the predictive value at different time intervals and supplemented the feasibility of using meta-regression to determine whether there is a declining trend in the predictive capacity of the model over time.

In clinical trials, model selection remains a noteworthy concern. Cox regression is the primary method in survival analysis, while logistic regression is predominantly used in non-survival analysis. Both models offer good interpretability. Balancing interpretability and accuracy in machine learning models is a key challenge in clinical practice. Generally, models with high interpretability, such as logistic regression, COX regression, decision trees, and the Fine & Gray model, raise concerns about accuracy. On the other hand, models with poor interpretability, like random forest, random survival forest, artificial neural networks, and deep learning, often achieve higher accuracy [64]. Due to the complex parameter adjustment rules of less.

interpretable models, accurately understanding the relationship between each indicator and the risk of death becomes challenging. Despite this, these models have significant advantages, especially in extracting predictive factors in image processing. However, in image analysis, models with poor interpretability still offer unique advantages [65]. In our study, we primarily considered common admission factors and some interpretable laboratory indicators. Therefore, we lean towards using models with better interpretability in this context, as they can more accurately reflect the relationship between clinical prediction indicators and the risk of death. This is crucial for providing enhanced visual support in developing clinical prevention policies or specific measures.

We evaluated the model we utilized using the PROBAST tool for quality assessment. However, the results of the assessment raised certain concerns, particularly regarding the stringent evaluation of statistical methods. We believe that the evaluation criteria for this tool may be overly strict. Firstly, the tool mandates a training set with EPV ≥ 20 and a validation set with a sample size exceeding 100, posing challenges for rare diseases. Secondly, considering the complexity of the data, we identify high dimensionality, collinearity, and data imbalance as primary concerns. Currently, it is challenging for medical research to publicly disclose raw data. Additionally, the tool requires an assessment of whether the predictive factors and their weight coefficients in the research align with the reported results, involving complex machine learning models, some with poor interpretability. As mentioned earlier, these models do not publicly disclose the weight coefficients of their factors, complicating the assessment of consistency. Therefore, we suggest that certain evaluation criteria in the PROBAST assessment tool may require updates in future research. In subsequent studies, we aim to utilize this tool to assess indicators of research rationality, ensuring a more rigorous approach to scientific research. Our research encompasses a larger number of studies and patients, enhancing the generalizability of the findings and providing more compelling evidence for evaluating the effectiveness of the machine learning model in predicting the risk of death in people with hemophilia.

### Advantages and limitations of the study

Our research offers initial evidence-based support for the effectiveness of machine learning in predicting HIV-related deaths. However, certain limitations need acknowledgment. Firstly, our systematic search for eligible original studies has its constraints. Despite our comprehensive summary of modeling variables, the diverse nature of these variables, coupled with limitations in the number of original studies, prevented us from reporting the predictive performance of machine learning models based on variable types. Additionally, the inclusion of model types is restricted, largely due to the prevalence of COX regression in death prediction. This dominance makes it challenging to incorporate other non-survival analysis models. Therefore, a careful explanation of this section of the results is imperative.

## Conclusions

In summary, this systematic review and meta-analysis have highlighted the valuable role of machine learning models in predicting the risk of death among HIV patients, particularly during long-term follow-up. The results indicate that these models exhibit robust predictive performance, supported by high c-index values in both the training and validation sets. Despite potential limitations, such as variations in research quality and heterogeneity, our findings endorse the practicality of employing machine learning models as effective tools for mortality prediction in HIV patients. This bears significant importance in enhancing risk assessment and clinical decision-making for the improvement of HIV care.

While this study emphasizes the commendable performance of machine learning models in predicting the risk of death in HIV/AIDS patients, future research could delve deeper into the external validation of these models across diverse patient populations and healthcare settings. Moreover, enhancing the predictive accuracy and clinical applicability of these models may be achieved by integrating additional clinical variables or biomarkers. Conducting longitudinal studies to assess the actual application and impact of these models on patient prognosis will also contribute to a thorough evaluation of their real efficacy.

This study presents compelling evidence supporting the effectiveness of machine learning models in predicting the risk of death in HIV/AIDS patients. The utilization of rigorous methods and the discovery of clinically relevant findings make these models promising tools for enhancing risk assessment and delivering tailored interventions for HIV care. To enhance the quality of life and extend the survival time of individuals with HIV who are at a high risk of premature mortality, it is recommended to prioritize the reinforcement of treatment follow-up, closely monitor medication adherence, and provide comprehensive family support.

## Data Availability

The original contributions presented in the study are included in the article.

## Abbreviations

PWH: Prediction of mortality in individuals with HIV
AIDS: Acquired Immune Deficiency Syndrome
HIV: human immunodeficiency virus
ART: antiretroviral treatment
